# Improved Traffic Sign Detection and Recognition Algorithm for Intelligent Vehicles

**DOI:** 10.3390/s19184021

**Published:** 2019-09-18

**Authors:** Jingwei Cao, Chuanxue Song, Silun Peng, Feng Xiao, Shixin Song

**Affiliations:** 1 State Key Laboratory of Automotive Simulation and Control, Jilin University, Changchun 130022, China; caojw18@mails.jlu.edu.cn (J.C.); songchx@126.com (C.S.); pengsilun@126.com (S.P.); xiaofengjl@jlu.edu.cn (F.X.); 2 College of Automotive Engineering, Jilin University, Changchun 130022, China; 3 School of Mechanical and Aerospace Engineering, Jilin University, Changchun 130022, China

**Keywords:** driving assistance, intelligent vehicles, traffic sign detection, convolutional neural network, traffic sign recognition

## Abstract

Traffic sign detection and recognition are crucial in the development of intelligent vehicles. An improved traffic sign detection and recognition algorithm for intelligent vehicles is proposed to address problems such as how easily affected traditional traffic sign detection is by the environment, and poor real-time performance of deep learning-based methodologies for traffic sign recognition. Firstly, the HSV color space is used for spatial threshold segmentation, and traffic signs are effectively detected based on the shape features. Secondly, the model is considerably improved on the basis of the classical LeNet-5 convolutional neural network model by using Gabor kernel as the initial convolutional kernel, adding the batch normalization processing after the pooling layer and selecting Adam method as the optimizer algorithm. Finally, the traffic sign classification and recognition experiments are conducted based on the German Traffic Sign Recognition Benchmark. The favorable prediction and accurate recognition of traffic signs are achieved through the continuous training and testing of the network model. Experimental results show that the accurate recognition rate of traffic signs reaches 99.75%, and the average processing time per frame is 5.4 ms. Compared with other algorithms, the proposed algorithm has remarkable accuracy and real-time performance, strong generalization ability and high training efficiency. The accurate recognition rate and average processing time are markedly improved. This improvement is of considerable importance to reduce the accident rate and enhance the road traffic safety situation, providing a strong technical guarantee for the steady development of intelligent vehicle driving assistance.

## 1. Introduction

With the rapid development of economy and technology in the modern society, automobiles have become an indispensable means of transportation in the daily travel of people. Although the popularity of automobiles has introduced considerable convenience to people, it has also caused a numerous traffic safety problems that cannot be ignored, such as traffic congestion and frequent road accidents. Traffic safety issues are largely caused by subjective reasons related to the driver, such as inattention, improper driving operation and non-compliance with traffic rules, and smart cars have become an effective means to eliminate these human factors [1,2,3,4,5]. Self-driving technology can assist, or even independently complete the driving operation, which is of remarkable importance to liberate the human body and considerably reduce the incidence of accidents [6,7]. Traffic sign detection and recognition are crucial in the development of intelligent vehicles, which directly affects the implementation of driving behaviors. Smart cars use a vehicle-mounted camera to obtain real and effective road traffic information; they can also recognize and understand traffic signs in real time in the actual road scenes to provide correct command output and good motion control for intelligent vehicles, which can remarkably improve the efficiency and safety of automatic driving [8,9,10]. Therefore, conducting an in-depth study on it is necessary.

The traffic sign recognition process generally includes two stages: traffic sign detection and recognition. However, in the daily natural conditions, the changes of light, the complex backgrounds and the aging of signs have caused many difficulties in accurately identifying traffic signs. With the rapid increase in computer running speed, many experts and scholars have focused on the traffic sign recognition process, which is mainly divided into traffic sign detection and recognition technologies [11,12,13,14]. Traffic sign detection technology is mainly based on inherent information, such as color, shape and texture features of traffic signs, and accurately extracts traffic sign candidate areas from the actual road scenes. Wang et al. [15] proposed a red bitmap method to detect traffic signs. Firstly, color segmentation of the detected images is performed, and then shape detection of the region of interest (ROI) based on edge information is conducted. This method achieved good detection results but was only applicable to red circular traffic signs, which had some limitations. Hechri et al. [16] used the template matching method to match the traffic signs. By setting the sliding window of the same size as the traffic signs, the useless parts of non-traffic signs in the current road scenes were removed. However, some signs had different shapes and sizes, and the road traffic environment was complex and changeable; thus, the real-time performance of this method was poor. Lillo-Castellano et al. [17] adopted the color segmentation method that combines HIS and LAB color spaces to enable detection of black, white and colorful traffic signs. Xiao et al. [18] proposed a traffic sign detection method combining HOG features and Boolean convolutional neural networks. This method can eliminate the error detection areas of candidate regions and achieve good detection results by connecting cascaded classifiers. Guan et al. [19] proposed a method for detecting traffic signs from mobile LiDAR point clouds and digital images. Traffic signs were detected from mobile LiDAR point clouds based on valid road information and traffic-sign size, and segmented by digital image projection, and the given images can be classified automatically after normalization. Traffic sign recognition technology is mainly used to analyze and classify the detected traffic signs and accurately obtain their actual meaning. Sun et al. [20] proposed a traffic sign classification method based on extreme learning machine (ELM), which is a supervised learning algorithm related to feedforward neural network. Only one hidden layer is observed; therefore, the parameters were few and the training time was short. The algorithm classified traffic signs according to the calculation results by selecting a certain proportion of features and obtained high recognition accuracy. Qian et al. [21] trained the traffic sign data by using the regional depth convolutional neural network (CNN) and the collected Chinese traffic sign dataset for identification test, which achieved a high accurate recognition rate. He et al. [22] proposed ResNet network based on the concept of residuals. By continuously learning the residuals, the network performance was considerably raised, and the recognition accuracy was further improved. Yuan et al. [23] adopted the traffic sign recognition combining Adaboost algorithm and support vector machine (SVM). The candidate recognition images were screened by the Adaboost and then classified by the SVM. The recognition accuracy was high, but the detection time was long. Kumar et al. [24] proposed a traffic sign detection method based on capsule network. The multi-parameter deep learning network structure can realize automatic feature extraction, which had good robustness and stability. The experimental results showed that the method had a conspicuous detection effect. Yuan et al. [25] proposed an end-to-end traffic sign detection method. The multi-feature fusion network structure can extract effective features for different size images, and then establishing a vertical space sequence attention module to obtain background information around the detected image, which also had prominent detection performance in complex road traffic environments. The research results show that many methods have improved the accurate recognition rate of traffic signs, but advantages and disadvantages still exist between the algorithms, which will be limited by various conditions. In the study of traffic sign detection technology, disturbances, such as bad weather conditions, changes in lighting conditions and fading of signage, will lead to an evident decline in the accuracy of traffic sign detection and poor environmental adaptability [26,27,28]. Moreover, recognition algorithms based on deep learning-based methodologies have a high accurate recognition rate, but some problems, such as high complexity of the algorithms and long processing time, exist. Meanwhile, the algorithms have high requirements on system hardware, and the structures of training models are complicated, thereby indicating the presence of some limitations [29,30,31,32]. Therefore, further improvement of the traffic sign detection and recognition algorithm is urgent.

In this study, an improved traffic sign detection and recognition algorithm for intelligent vehicles is proposed. Firstly, the HSV color space is used for spatial threshold segmentation, and traffic signs are effectively detected based on the shape features. Secondly, the model is considerably improved on the basis of the classical LeNet-5 convolutional neural network model by using Gabor kernel as the initial convolutional kernel, adding the batch normalization (BN) processing after the pooling layer and selecting the Adam method as the optimizer algorithm. Finally, the traffic sign classification and recognition experiments are conducted based on the German Traffic Sign Recognition Benchmark (GTSRB). The favorable prediction and accurate recognition of traffic signs are achieved through the continuous training and testing of the network model. According to the analysis of experimental results and performance comparison with other algorithms, the comprehensive performance of the algorithm is evaluated.

The rest of this paper is organized as follows: In Section 2, the HSV color space is used for spatial threshold segmentation, and traffic signs are effectively detected based on the shape features. In Section 3, the classic LeNet-5 CNN model is further improved. In Section 4, the experiments on traffic sign classification and recognition based on the GTSRB are conducted and analyzed, and the performance of algorithms are compared. In Section 5, conclusions and suggestions for possible future work are outlined.

## 2. Traffic Sign Detection

The road traffic images are captured by vehicle-mounted cameras installed on the smart cars, and the traffic sign detection aims to extract the interested traffic sign regions from the current road traffic images sufficiently. However, in different external conditions, the qualities of the acquired images are uneven, and these qualities must be effectively detected following the inherent characteristics of traffic signs, such as color and shape. In this section, it mainly includes two parts: traffic sign segmentation based on the color space and traffic sign detection based on shape features.

### 2.1. Traffic Sign Segmentation Based on the HSV Color Space

**Figure 1:**
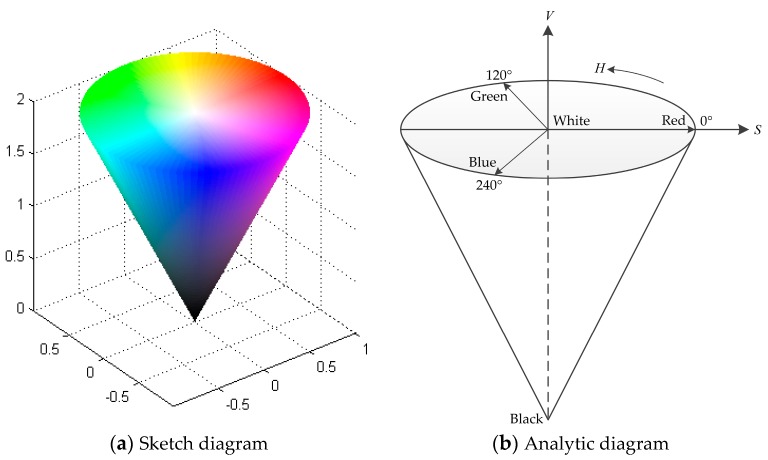
The HSV color space.

Color is an important feature of traffic sign, and traffic sign can be quickly located by color segmentation. Compared with RGB color space and HSI color space, the HSV color space has a faster detection speed, less affected by illumination, and has a preferable segmentation advantage. Figure 1 shows the HSV color space converted from the RGB color space. It represents the points in the RGB color space by an inverted cone, where H is the hue, S is the saturation and V is the value. 

H indicates the color change of the image. The position of the spectral color is represented by the angle, and different color values correspond to different angles. Red, green and blue are 120° apart, that is, 0°, 120° and 240°, respectively. S denotes the proportion of the current color purity to the maximum purity with the maximum value of 1 and the minimum value of 0. V represents the brightness change of the image. The maximum value is 1 in white and the minimum value is 0 in black. In the HSV color space, given that V is a fixed value set and H and S are highly unrelated, the HSV color space has good illumination adaptability when the illumination conditions change, and its computational complexity is small, which are conducive to the color space threshold segmentation.

**Figure 2:**
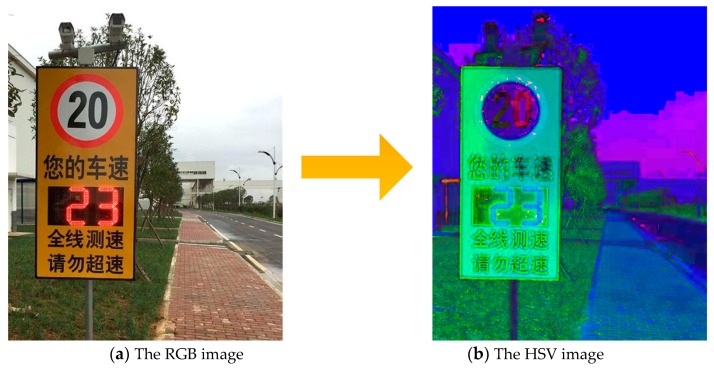
Converting the RGB image to the HSV image.

The conversion of an RGB to an HSV image is shown in Figure 2.

**Figure 3:**
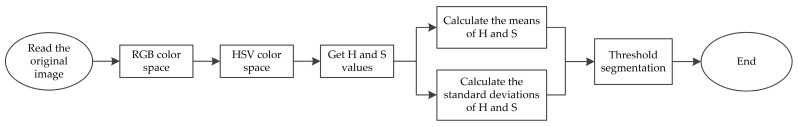
The color space threshold segmentation step diagram.

Color space threshold segmentation is required after conversion to the HSV color space. Figure 3 shows the color space threshold segmentation step diagram.

Common traffic signs mainly include red, yellow and blue colors. In order to meet the target requirements of real-time color segmentation, it is necessary to determine the corresponding threshold range. Through multiple test experiments, the three-channel threshold segmentation ranges of three colors are obtained on the premise of ensuring good segmentation effects, as shown in Table 1.

**Figure 4:**
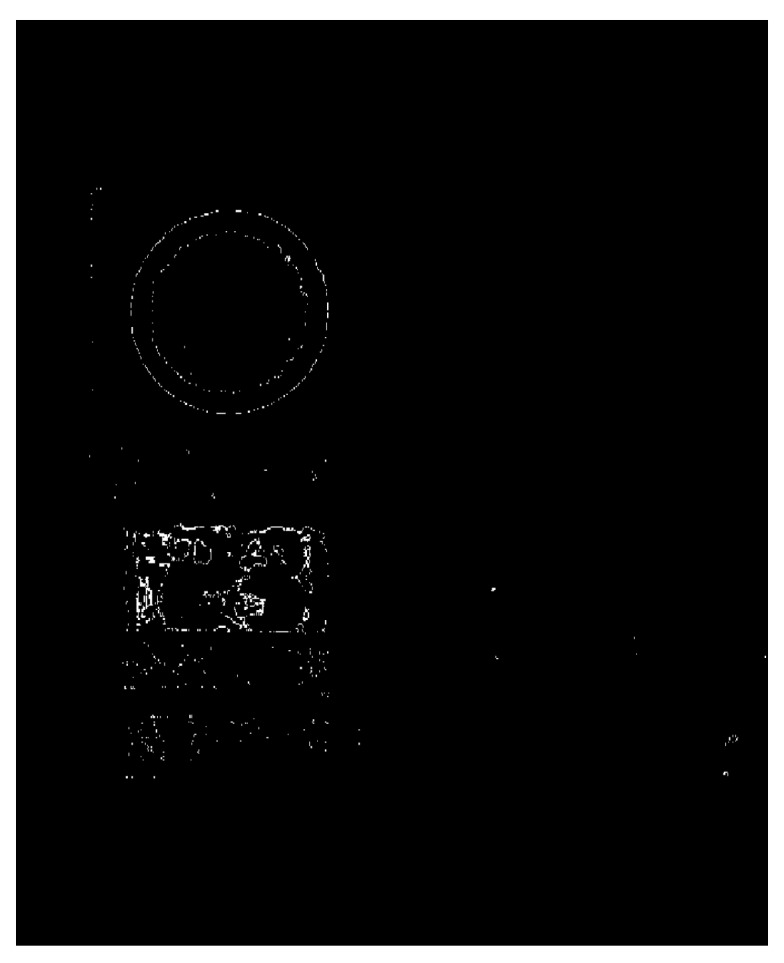
The threshold rough segmentation image.

In the process of threshold segmentation, the pixels within the set threshold range are set to white, otherwise they are set to black, and the image is completely binarized. Since the traffic sign in the original picture is red, the obtained threshold coarse segmentation image only displays red. Figure 4 presents the threshold rough segmentation image.

### 2.2. Traffic Sign Detection Based on the Shape Features

**Figure 5:**
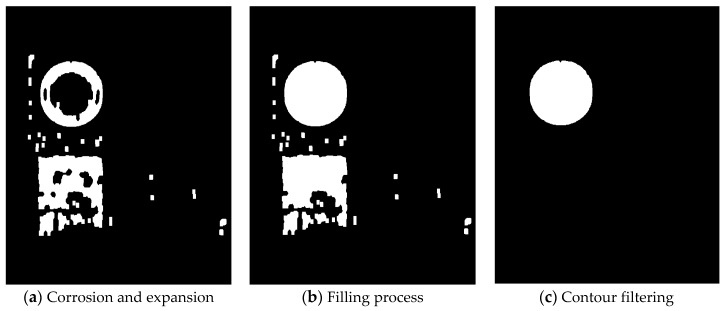
The morphological processing for binary images.

In the actual road scenes, traffic signs do not exist independently. Colorful clothes of pedestrians and colored billboards are likely to be consistent with the color of traffic signs, thereby resulting in some useless interference to the binary image with threshold coarse segmentation. Therefore, filtering these interferences is necessary to achieve effective detection of the ROI. Figure 5 illustrates the morphological processing for binary images.

Firstly, the binary image is processed by image corrosion and expansion. Some isolated useless pixels often exist on the edge of the image, and these pixels can be effectively removed by corrosion. Meanwhile, expansion aims to enlarge the area of the ROI. The combination of them can filter out some subtle interference, thereby producing prominent shape characteristics of traffic signs. 

The filling process is then conducted. The traffic signs may be discolored, damaged and blocked by some obstacles in the actual road scenes, and the ROI cannot be completely displayed. The filling process can help complete and visualize the contours of traffic signs. 

Finally, the effective detection of traffic signs is realized. Some large irregular interference areas still exist in the segmented image after the filling process and thus need to be filtered. Contour filtering is conducted by the contour analysis of connected area. This are in the image is a set with all the same pixel points. The circumference and area of the contours of all connected areas are calculated and then compared with the standard circular mark. The contours that meet the requirements are retained; otherwise, they are discarded. Similarly, this method is equally applicable to the traffic sign detection of triangle, rectangle and other shapes. The remaining part of the segmented image after contour filtering corresponds to the detected traffic sign.

## 3. Improved LeNet-5 CNN Model

Traffic sign recognition is based on existing dataset resources and uses effective classification algorithm to recognize detected traffic signs and feedback to smart cars accurately in real time. CNN extracts features directly from the input detection image and outputs the classification results via the trained classifier based on image features. This condition indicates that CNN has good graphic recognition performance. Furthermore, CNN does not need to extract features manually. The sensory cognitive process of human brains can be well simulated via forward learning and feedback mechanism, thereby gradually improving the ability of traffic sign classification and recognition [33,34]. In this section, the shortcomings of the classical LeNet-5 network model are analyzed, and the model is considerably improved to further expand the outstanding advantages of CNN in graphics recognition.

### 3.1. Deficiency Analysis of Classical LeNet-5 Network Model

Professor Yann Lecun proposed the LeNet-5 network model in 1998, which was mainly used for digital recognition. The LeNet-5 network model consists of seven layers, including two convolutional layers, two pooling layers, two fully-connected layers and one output layer. The input image size is 32 × 32, and the output is a 10-dimensional classification vector, which can identify numbers from 0 to 9 [35,36].

The classic LeNet-5 network model has good classification and recognition effects for a single target. However, in the traffic signs recognition training, it is difficult to ensure a high enough accurate recognition rate, the training network cannot converge, and the recognition efficiency of the network decreases dramatically.

Analysis and summary of the root causes of these problems show the following:

(1) The interference background in the traffic sign training image is much more complicated than that in a single digital image. The original convolutional kernel does not perform well in feature extraction. Consequently, the extracted features cannot be properly used for the accurate classification of the subsequent classifier.
(2) Different kinds of traffic sign training images exist, and the number of datasets is large. Gradient dispersion easily occurs during network training, and the generalization ability is significantly markedly reduced.
(3) The size of the ROI in the input traffic sign training image varies, and the effective features obtained by the current network model are insufficient to meet the target requirements of accurate traffic sign recognition.
(4) The learning rate and the iterations number of the training network are not adjusted accordingly, and the relevant parts are rationally optimized, thereby resulting to the emergence of the over-fitting phenomenon during training.

### 3.2. Improved LeNet-5 Network Model

#### 3.2.1. Image Preprocessing

The ROI in the traffic sign training image is not 100% in the center of the image, and some edge background information is included around the traffic sign. With the change of illumination conditions, these useless interference areas will increase the influence on traffic sign recognition, thereby undoubtedly raising the computational complexity of the training network and the misrecognition rate of traffic signs. Therefore, image preprocessing is necessary.

Image preprocessing mainly includes the following three stages:

(1) Edge clipping. Edge cropping is a particularly important step in the image preprocessing. Some background parts in the edge are not related to traffic signs, and these parts can account for approximately 10% of the entire image. The bounding box coordinates are used for proportional cropping to obtain the ROI. The removal of the interference region helps to reduce redundant information and speed up the network training.
(2) Image enhancement. The recognition effects of the same type of traffic signs in the training network under different illumination conditions are significantly different. Therefore, reducing or removing the noise interference caused by the light change via image enhancement is necessary. Direct grey-scale conversion method is used to adjust the grey value of the original image using the transformation function, which presents clear details of the ROI and demonstrates a blurred interference area. Thus, this method effectively improves the image quality and reduces the computational load of the training network.
(3) Size normalization. The same type of traffic signs may have different sizes. The different sizes of training images may have different feature dimensions during the CNN training process, which leads to difficulties in the subsequent classification and recognition. In this paper, the image is uniformly normalized in size, and the normalized image size is 32 × 32.

#### 3.2.2. Improved LeNet-5 Network Model

**Figure 6:**
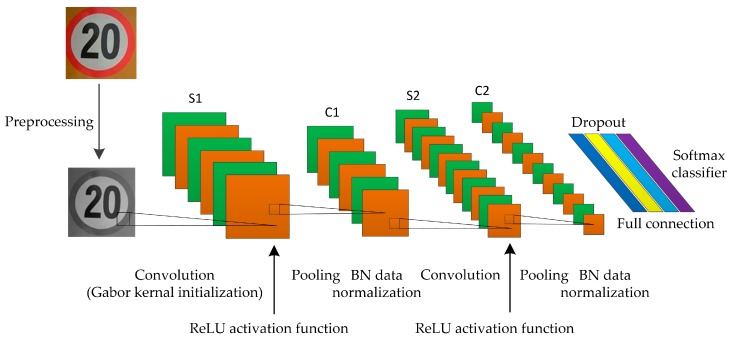
The improved LeNet-5 network model structure.

The LeNet-5 network model has been considerably improved due to the shortcomings of the classic model in traffic sign recognition. Figure 6 shows the improved LeNet-5 network model structure.

The improvement of LeNet-5 network model includes the following five aspects.

(1) The Gabor kernel is used as the initial convolutional kernel between the input layer and the first convolutional layer. In the actual road scenes, the change of light, the damage of traffic signs, and the obstruction of obstacles will affect the quality of the training image. Nonetheless, Gabor wavelet can solve such problems commendably. The Gabor wavelet is insensitive to changes in light; therefore, it has good adaptability to light. Furthermore, it has superior scale and direction selection characteristics that are sensitive to the edges of the training image.

The two-dimensional Gabor filter is a band-pass filter whose impulse response function is as follows:

(1) $$g(x,y,f,\theta )=\frac{1}{2\pi \sigma_{x}\sigma_{y}}\exp(-\frac{k_{1}^{2}}{2\sigma_{x}^{2}}-\frac{k_{2}^{2}}{2\sigma_{y}^{2}})\exp\left(i(f_{x}x+f_{y}y)\right)$$

(2) $$k_{1}=x\cos\theta +y\sin\theta ,k_{2}=-x\sin\theta +y\cos\theta$$

where *f* is the center frequency of the bandwidth; θ is the spatial direction whose value ranges [0,π]; $\sigma_{x}$ and $\sigma_{y}$ are the standard deviations in the *x* and *y* directions, respectively; $f_{x}=f\cdot \cos\theta$ and $f_{y}=f\cdot \sin\theta$ are both frequencies in space.

When $\sigma_{x}=\sigma_{y}$, the Equation (1) can be converted to:

(3) $$g(x,y,f,\theta )=\frac{1}{2\pi \sigma^{2}}\exp(-\frac{x^{2}+y^{2}}{2\sigma^{2}})\exp\left(i(f_{x}x+f_{y}y)\right)$$

Given that Gabor filters vary in different scales and directions, the mean value of Gabor kernels in different directions at the same scale is taken as the initial convolutional kernel in this paper.

(2) After each pooling layer, the BN is added for data normalization. In the deep learning network model, as the number of training increases, the hidden layer gradient near the output layer expands and the parameter updating accelerates. Meanwhile, the hidden layer gradient near the input layer shows the opposite; that is, presenting a state of random distribution called gradient dispersion, while BN data normalization can effectively solve this problem.

The BN data normalization is as follows:

Input: Mini-batch input $x:B=\left\{x_{1},\ldots ,m\right\}$.

Output: Normalized network response $\left\{y_{i}=BN_{\gamma ,\beta }(x_{i})\right\}$.

1. The mean of training batch data:
  (4) $$\mu_{B}=\frac{1}{m}\sum_{i=1}^{m}x_{i}$$
2. The variance of training batch data:
  (5) $$\sigma_{B}^{2}=\frac{1}{m}\sum_{i=1}^{m}{\left(x_{i}-\mu \right)}^{2}$$
3. Normalization:
  (6) $${\hat{x}}_{i}=\frac{x_{i}-\mu_{B}}{\sqrt{\sigma_{B}^{2}+\varepsilon }}$$
  where ε is the minimum positive number used to avoid division by 0.
4. Scale transformation and offset:
  (7) $$y_{i}=\gamma {\hat{x}}_{i}+\beta$$
5. The learning parameters γ and β are returned.

The BN data normalization results in the output mean of 0 and the variance of 1. These results are beneficial to the non-linear expression of the model and provides consistent output distribution with the real data distribution. The application of deep network models is not only appropriate but also has good effects in shallow network models.

(3) The ReLU function is selected as the activation function. Compared with the traditional Sigmoid and Tanh functions, the ReLU function is simple in calculation but effectively solves the gradient disappearance and explosion problem of the two functions. By making a part of the neuron output to 0, the network can be sparse, which helps reduce computational complexity and accelerate network convergence. Therefore, this function performs well in deep network training.

(4) The Adam method is chosen as the optimizer algorithm. This method is an extended first-order optimization algorithm based on the stochastic gradient descent method, which can dynamically adjust the learning rate of related parameters by using the moment estimation of the gradient. After the offset correction, the Adam method can control each iterative learning rate within a certain range, thereby ensuring a smooth updating of the network parameters.

The first moment of the gradient is as follows:

(8) $$m_{t}=\beta_{1}m_{t-1}+(1-\beta_{1})g_{t}$$

The second moment of the gradient is as follows:

(9) $$v_{t}=\beta_{2}v_{t-1}+(1-\beta_{2})g_{t}^{2}$$

where $\beta_{1}$ and $\beta_{2}$ are the attenuation factors, and $g_{t}$ is the gradient value of the loss function at time *t*.

The first moment deviation estimate of the gradient is as follows:

(10) $${\hat{m}}_{t}=\frac{m_{t}}{1-\beta_{1}^{t}}$$

The second moment deviation estimate of the gradient is as follows:

(11) $${\hat{v}}_{t}=\frac{v_{t}}{1-\beta_{2}^{t}}$$

The gradient update formula of the Adam method is as follows:

(12) $$\theta_{t}=\theta_{t-1}-\frac{\eta }{\sqrt{{\hat{v}}_{t}}+\varepsilon }{\hat{m}}_{t}$$

where η is the initial learning rate.

The Adam method is computationally efficient and requires less memory space. Thus, this method is suitable for solving optimization problems with large-scale data and parameters. The Adam method can effectively solve the problems of learning rate disappearance, slow convergence and large fluctuation of loss function in the optimization process, thereby possessing a good convergence mechanism.

(5) The dropout is added to the fully-connected layers. It temporarily discards half of the data flowing through the network by discarding some neurons. Before the new round of data iteration, the original fully connected model is restored, and then some neurons are randomly removed. The dropout can considerably reduce the amount of network computation, help weaken the joint adaptability between neuron nodes, enhance the generalization ability of the training model and play a regularization role to a certain extent to prevent over-fitting problems.

Table 2 lists the parameter settings of the improved LeNet-5 network model.

In this paper, the classical LeNet-5 network model is improved in many aspects and multiple levels. Considering the different interference conditions that may occur in the actual road scenes, the improved LeNet-5 network model integrates multiple advantages into one, thereby fostering strengths and avoiding weaknesses and complementing each other. The robustness and stability of the training network are considerably enhanced, and the overall convergence speed is improved, thereby further enhancing the performance levels of traffic sign classification and recognition.

## 4. Traffic Sign Recognition Experiment

### 4.1. Experimental Environment

Software environment: Windows 10 64-bit operating system, JetBrains PyCharm 2019.1.1, TensorFlow 1.13.1, Python 3.7.0 64-bit.

Hardware environment: Intel (R) Core (TM) i5-6500 CPU@3.20GHz processor, 8.00 GB memory, 2 TB mechanical hard disk.

### 4.2. Traffic Sign Recognition Experiment

#### 4.2.1. Traffic Sign Dataset

This paper uses the German Traffic Sign Recognition Benchmark (GTSRB), which was presented at the 2011 International Joint Conference on Neural Networks (IJCNN). The internal traffic signs are collected from the real road traffic environment in Germany, and it has become a common traffic sign dataset used by experts and scholars in computer vision, self-driving and other fields. The GTSRB comprises 51,839 images, which are divided into training and testing sets. A total of 39,209 and 12,630 images are provided in the training and testing sets, accounting for approximately 75% and 25% of the whole, respectively. Each image contains only one traffic sign, which is not necessarily located in the center of the image. The image size is unequal; the maximum and smallest images are 250 × 250 and 15 × 15 pixels, respectively [37,38].

**Figure 7:**
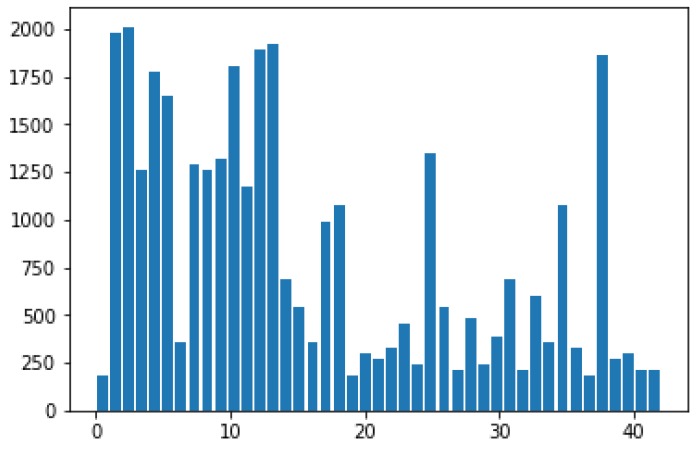
The number of 43 classes of traffic signs.

**Figure 8:**
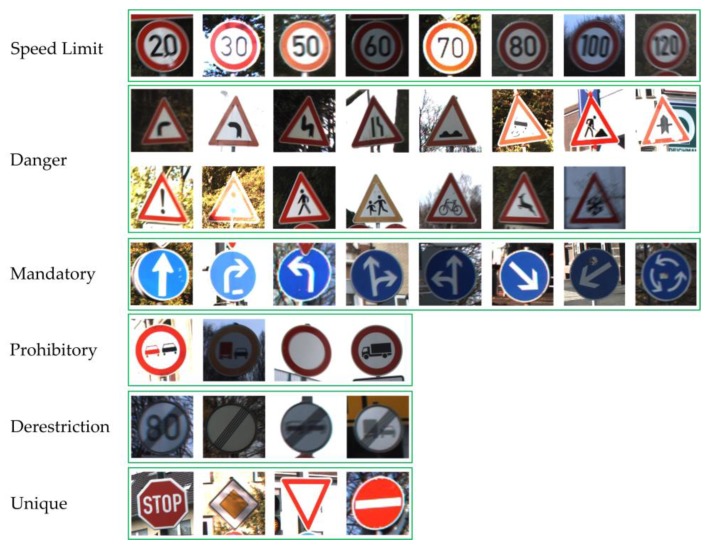
Six categories of traffic signs sample images.

The traffic sign images in GTSRB are taken from the video captured by the vehicle-mounted camera. As shown in Figure 7, GTSRB includes 43 classes of traffic signs, and the number of different types of traffic signs varies. Each type of traffic sign corresponds to a catalogue, which contains a CSV file annotated with a class label and a single image of multiple tracks (each track includes 30 images). In accordance with the different instruction contents, GTSRB can also be divided into six categories: speed limit, danger, mandatory, prohibitory, derestriction and unique traffic signs as shown in Figure 8. The same type of traffic signs include different resolutions, illumination conditions, weather conditions, occlusion degree, tilt levels and other images, making the dataset more in line with the actual road scenes.

**Figure 9:**
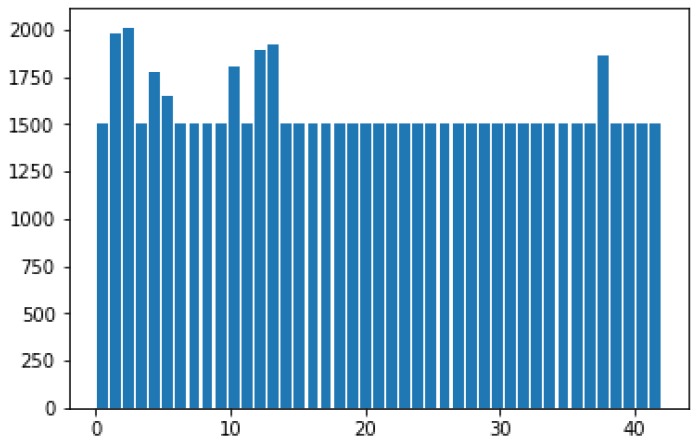
The number of 43 classes of traffic signs after generating the artificial dataset.

After image preprocessing, an artificial dataset must be generated for GTSRB. Given that the number of different types of traffic signs in GTSRB varies, this condition easily causes the imbalance of sample data. Different types of traffic signs have evident differences during classification and recognition, which affect the generalization of the entire network model. Generating an artificial dataset aims to construct a new artificial sample by randomly sampling from the value space of each attribute feature of the same sample type. Therefore, the number of different kinds of traffic signs is as equal as possible to solve the problem of sample data imbalance. After generating the artificial dataset, the 43 classes of traffic signs are shown in Figure 9.

#### 4.2.2. Traffic Sign Classification and Recognition Experiment

Traffic sign classification and recognition experiment can be divided into two stages, namely, the network training and testing stages. In the network training stage, the training set samples of GTSRB are taken as input. By performing thousands of network iterations, parameters, such as network weights and offsets, are continuously updated on the basis of forward learning and back propagation mechanisms until the loss function is reduced to the minimum, thereby classifying and predicting traffic signs. In the network testing stage, the testing set samples of GTSRB are inputted into the trained network model to test the accurate recognition rate of the training network.

**Figure 10:**
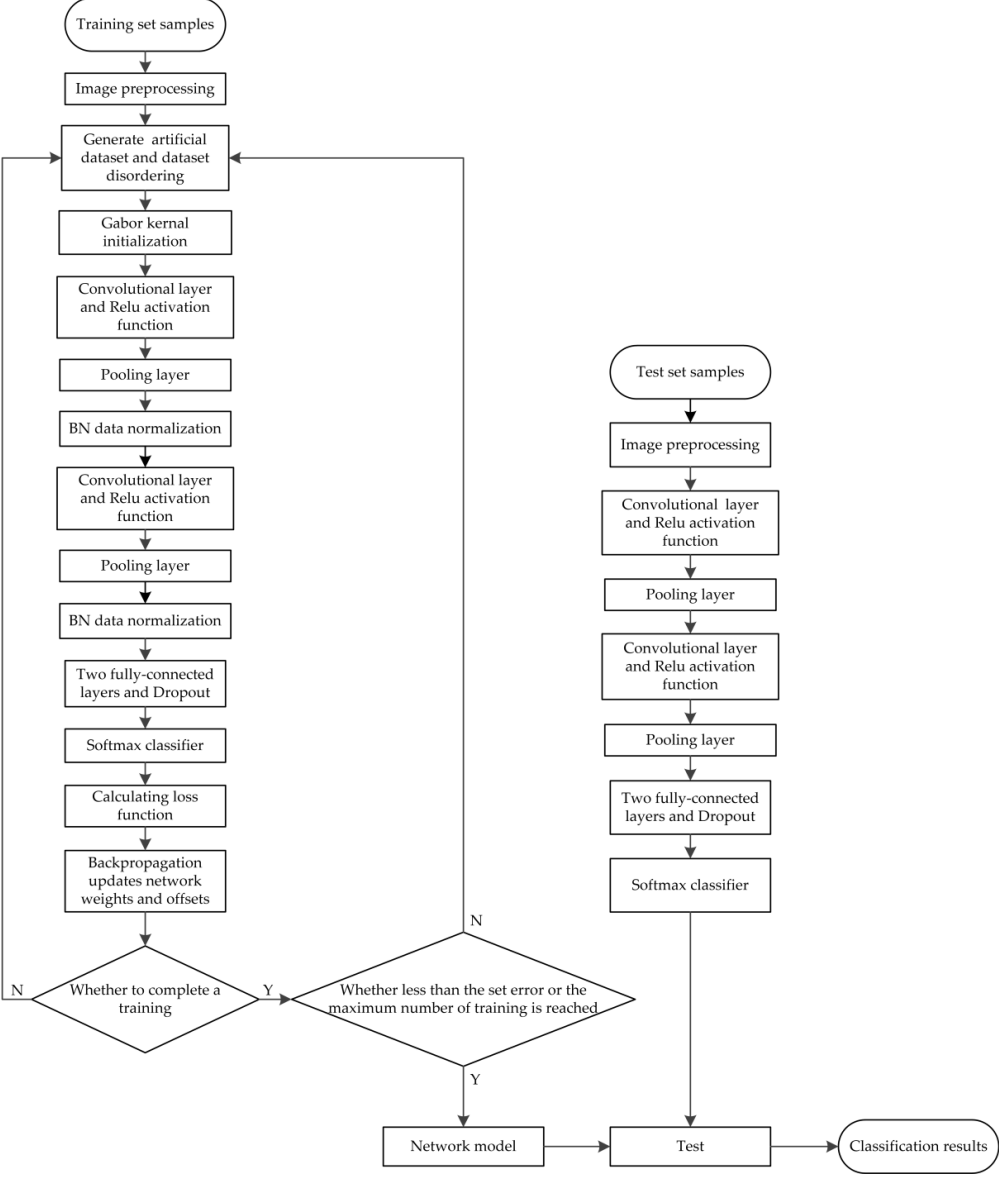
The flow chart of the entire traffic sign classification and recognition experiment.

Figure 10 shows the flow chart of the entire traffic sign classification and recognition experiment.

The basic steps of the network training stage are as follows.

(1) The training set samples are preprocessed, the artificial dataset is generated and the dataset order is disrupted.
(2) The Gabor kernel is used as the initial convolutional kernel, and the convolutional kernel size is 5 × 5, as activated by the ReLU function.
(3) The training set samples are forwardly propagated in the network model, and a series of parameters are set. The BN is used for data normalization after each pooling layer, and the Adam method is used as the optimizer algorithm. The parameters are set as follows: $\beta_{1}=0.9$, $\beta_{2}=0.999$, η=0.001 and $\varepsilon =1\times 10^{-8}$. The dropout parameter is set to 0.5 in the fully-connected layers, and the Softmax function is outputted as a classifier.
(4) The gradient of loss function is calculated, and the parameters, such as network weights and offsets, are updated on the basis of the back-propagation mechanism.
(5) The error between the real and the predicted value of the sample is calculated. When the obtained error is lower than the set error or reaches the maximum number of training, training is stopped and step (6) is executed; otherwise, step (1) is repeated for the next network iteration.
(6) The classification test is conducted in the network model. The subordinate categories of traffic signs in the GTSRB are predicted and compared with the real categories. The classification prediction results of traffic signs are counted, and the correct prediction rate is calculated.

The basic steps of the network testing stage are as follows.

(1) Several images are randomly selected from the testing set samples, and the images are inputted into the trained network model after preprocessing.
(2) The recognition results are outputted through the network model, thereby showing the meaning of traffic signs with the highest probability.
(3) The output results are compared with the actual reference meanings, and the statistical recognition results are obtained.
(4) All the sample extraction images are completely tested, and the accurate recognition rate of traffic signs is calculated.

**Figure 11:**
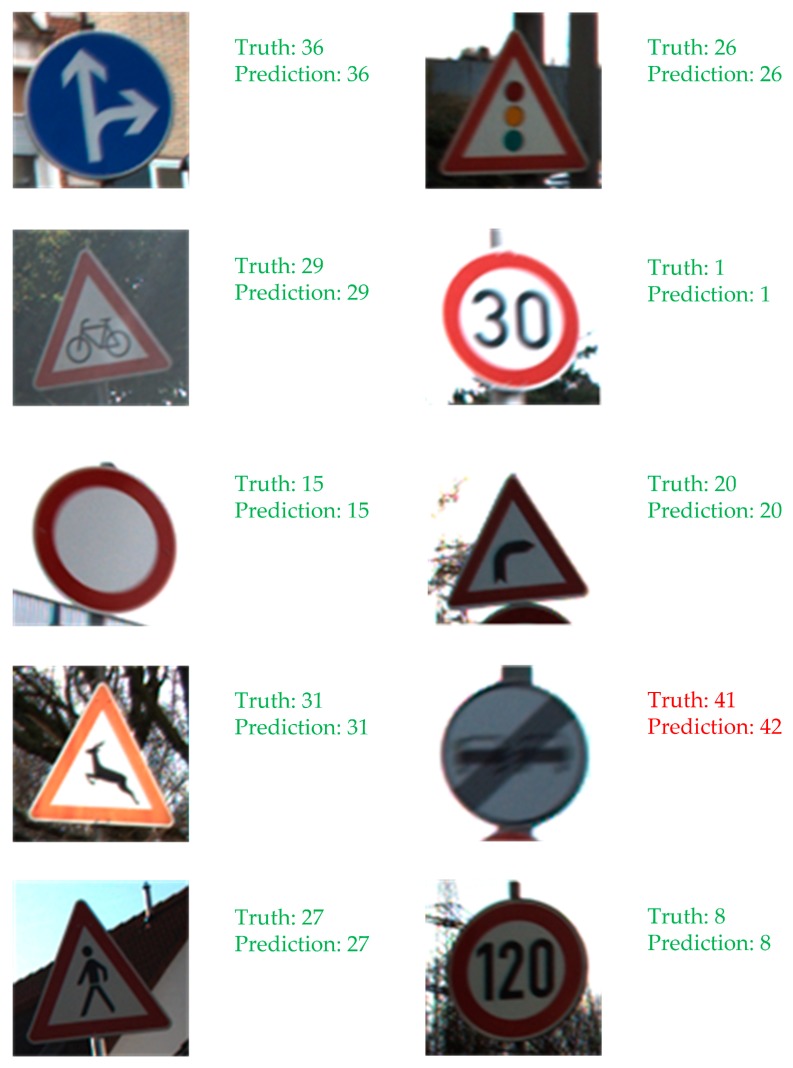
The classification prediction results of some sample images in the network training stage.

Figure 11 shows the classification prediction results of some sample images in the network training stage.

**Figure 12:**
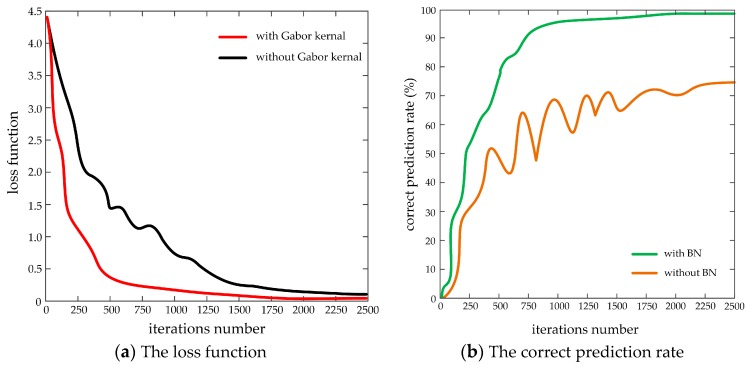
The dynamic change curve of relevant parameters in the network training stage.

Figure 12 presents the dynamic change curve of relevant parameters in the network training stage, in which, (a) indicates the dynamic contrast curve of loss function with iteration number in the case of Gabor and non-Gabor kernels, (b) shows the dynamic contrast curve of correct prediction rate with iteration number in the case of BN data normalization and non-BN data normalization.

As shown in Figure 12a, in the improved Lenet-5 network model, with the deepening of the network iterations, the loss function corresponding to the Gabor kernel initialization is much falling faster than that without the Gabor kernel initialization, and drops smoothly to 0. The reason is that the Gabor filter can extract effective target contour information and remove useless image noise, thereby effectively avoiding over-fitting of the training data and reducing the computational complexity, and further enhance the robustness and adaptability of the network model. Without the Gabor filter, the training network can easily fall into the local optimal solution, which makes the updating of network parameters such as weights and offsets become slower. It can be seen from Figure 11 and Figure 12b that a good sample image classification prediction effect is achieved in the network training stage, and the correct prediction rate using BN data normalization increases with iteration number and the highest value can reach about 99.82%. When BN data normalization is not used, the correct prediction rate has a large fluctuation and the highest value is only about 75%. The reason is that after adding BN data normalization processing, not only can the gradient dispersion phenomenon be effectively avoided, but also the convergence speed of the training model can be accelerated, the training model is more stable, and the generalization ability can be considerably enhanced.

**Figure 13:**
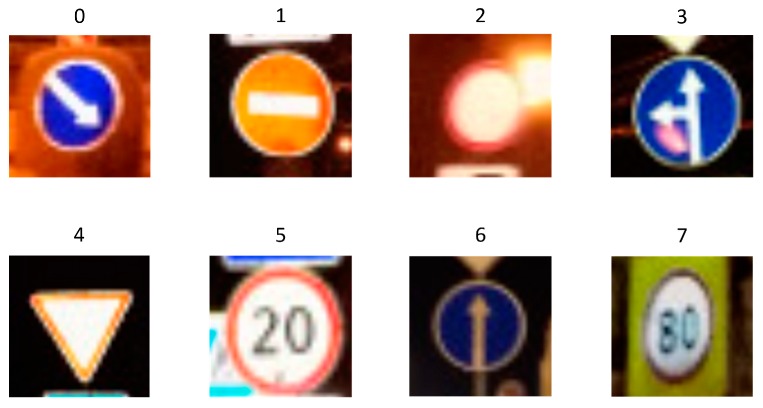
The auto-numbered traffic sign test images.

In the network testing stage, eight different traffic sign test images are randomly selected from the testing set samples and numbered automatically. Figure 13 shows the auto-numbered traffic sign test images.

**Figure 14:**
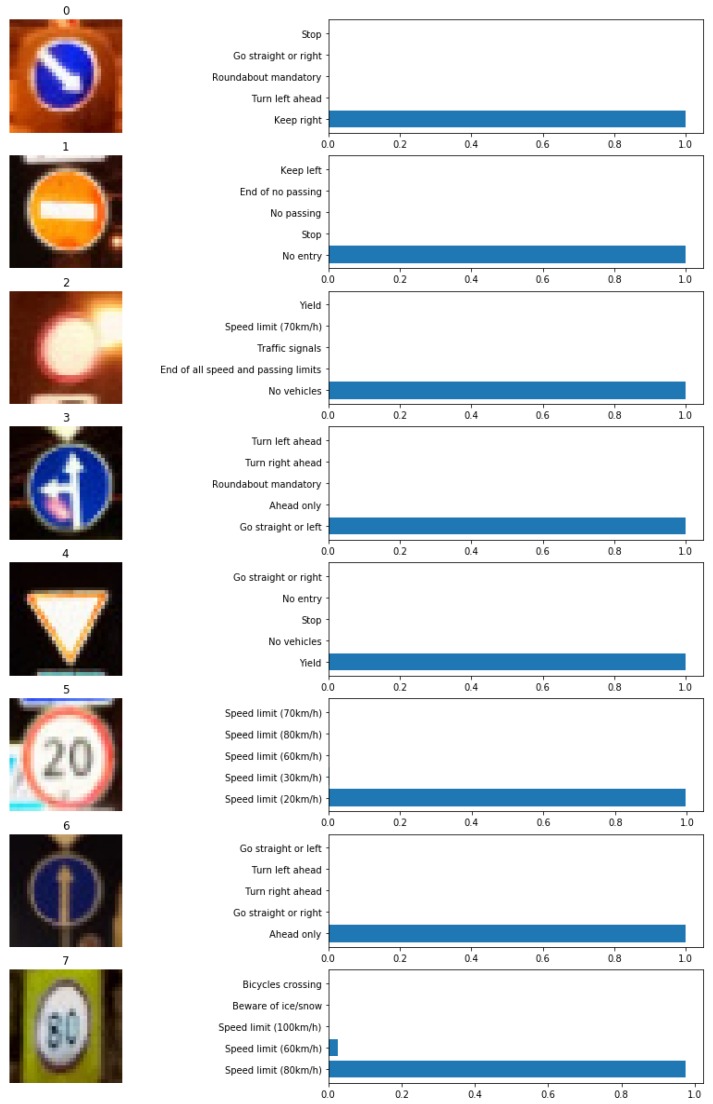
The recognition results of traffic sign test images in the network testing stage.

The traffic sign test images are inputted into the trained improved LeNet-5 network model for classification and recognition. For each test image, the traffic sign indicated by the first five probabilities are outputted, and the maximum probability is selected as the recognition result and compared with the actual reference meaning. Figure 14 shows the recognition results of traffic sign test images in the network testing stage.

It can be seen intuitively from the Figure 14 that the maximum probability recognition results of the eight traffic sign test images are completely consistent with their true meaning, and all of them have achieved effective recognition with an absolute probability close to 100%. The recognition results in the network testing stage show that the trained improved LeNet-5 CNN model has excellent classification and recognition ability, strong anti-jamming ability and high accuracy recognition rate for traffic sign dataset with different backgrounds and interferences, thereby reflecting admirable robustness and accuracy.

#### 4.2.3. Statistics and Analysis of Experimental Results

A total of 1000 test images are randomly selected respectively from six categories of traffic signs classified roughly in the GTSRB for classification and recognition to test the comprehensive recognition performance of the improved LeNet-5 network model for different types of traffic signs. Table 3 lists the classification and recognition test results of six categories of traffic signs.

As shown in Table 3, TP (True Positive) is the number of test images in which traffic signs are correctly recognized, and FN (False Negative) is the number of test images in which traffic signs are misrecognized and missed. In the traffic sign classification and recognition test experiments, the unique traffic signs perform best in the test results due to the advantages of fixed contours and distinctive features. The accuracy recognition rate reaches 100.00%, and the average processing time per frame is 4.7 ms. The derestriction traffic signs perform worst in the test results due to disadvantages of consistent contours and similar features. However, the accurate recognition rate also reaches 99.40%, and the average processing time per frame is 6.4 ms. In total, the average accurate recognition rate of six categories of traffic signs reaches 99.75%, and the average processing time per frame is 5.4 ms. On this basis, the improved LeNet-5 network model has excellent image recognition performance, and the proposed traffic sign recognition algorithm has good real-time performance and adaptability.

By sorting and analyzing false or missed test images, the majority of these images are caused by extremely dark light, extremely low resolution, motion blur and excessive tilt. In the future, complex network models must be built aimed at these problems, and additional abundant datasets must be adopted to facilitate the accurate recognition of additional traffic signs with interference factors by CNN. In this manner, the inclusiveness and stability of traffic sign recognition algorithm are continuously improved.

### 4.3. Performance Comparison of Recognition Algorithms

The proposed algorithm is compared with other algorithms adopted in other literature to verify the performance of traffic sign recognition algorithms. Table 4 lists the comparison of statistics in algorithm performance based on the GTSRB dataset.

In the performance comparison experiment, the proposed algorithm and other literature all conducted relevant traffic sign recognition test experiments based on the GTSRB dataset. In reference [39], a traffic sign extraction method based on oriented gradient maps and the Karhunen-Loeve transform was adopted, which achieved good test results by reducing the number of attributes and combining multilayer perceptron. Compared with other algorithms, although the average processing time of this algorithm was relatively short, the accurate recognition rate was the lowest. Therefore, this algorithm is more likely to cause false or missed recognition in the actual road scenes than other algorithms. In reference [40], iterative nearest neighbors-based linear projection was combined with iterative nearest-neighbor classifier. Multiple HOG features were used for detection, and sparse representations were adopted for classification, thereby achieving good recognition performance. Compared with literature [39], although the accurate recognition rate was considerably improved, the average processing time was excessively long, and real-time performance was poor when applied to actual road scenes. In reference [41], a traffic sign recognition method based on the histogram of oriented gradients was utilized. By combining Gaussian filter and histogram equalization for effective image preprocessing, using principal component analysis for dimensionality reduction, and a good classification accuracy was achieved by using a kernel extreme learning machine (K-ELM) classifier, while the average processing time was also further shortened. In reference [42], the weighted multi-CNN was trained by a new training method, and good recognition accuracy was obtained. Although the running environment of the algorithm included GPU and CPU, the average processing time was still relatively long. Deep learning-based methodologies can still be further improved because of the complex structure of the training model, the large amount of calculation, the long training time and the poor real-time performance. Compared with the aforementioned literature, the proposed algorithm has the best overall performance when using the same dataset. The accurate recognition rate reaches 99.75%, and the average processing time per frame is 5.4 ms. The generalization ability and recognition efficiency of the network model are also remarkably improved. In terms of performance improvement, evident advantages are observed. The fully improved traffic sign recognition accuracy is conducive to considerably enhancing the driving safety of intelligent vehicles in the actual driving environments. Meanwhile, the fully shortened average processing time is conducive to meeting the real-time target requirements of intelligent vehicles in the actual driving environments effectively. Thus, this study contributes to further improving the technical level of intelligent vehicle driving assistance.

## 5. Conclusions

In this study, an improved traffic sign detection and recognition algorithm is proposed for intelligent vehicles. Firstly, the HSV color space is used for spatial threshold segmentation, and traffic signs are effectively detected based on the shape features. Secondly, this model is considerably improved on the basis of the classical LeNet-5 CNN model by using Gabor kernel as the initial convolutional kernel, adding the BN processing after the pooling layer, selecting Adam method as the optimizer algorithm. Finally, the traffic sign classification and recognition experiments are conducted based on the GTSRB. The favorable prediction and accurate recognition of traffic signs are achieved through the continuous training and testing of the network model. The experimental results show that the accurate recognition rate of traffic signs reaches 99.75%, and the average processing time per frame is 5.4 ms. The proposed algorithm has more admirable accuracy, better real-time performance, stronger generalization ability and higher training efficiency than other algorithms. The accurate recognition rate and average processing time are significantly improved.

From the viewpoint of traffic sign recognition accuracy and algorithm time-consuming, the proposed traffic sign detection and recognition algorithm has remarkable advantages. Considerably enhancing the driving safety of intelligent vehicles in the actual driving environments and effectively meeting the real-time target requirements of smart cars are conducive. Furthermore, a strong technical guarantee is provided for the steady development of intelligent vehicle driving assistance. In the future, the inclusiveness and anti-error recognition of the traffic sign recognition algorithm can be further optimized and improved to exploit the overall performance of the algorithm.

## Figures and Tables

**Table 1:** HSV color space threshold segmentation ranges.

Color	*H*	*S*	*V*
Red	H>0.90|H<0.10	S>0.40	V>0.35
Yellow	0.50<H<0.70	S>0.40	V>0.40
Blue	0.09<H<0.18	S>0.35	V>0.40

**Table 2:** The parameter settings of the improved LeNet-5 network model.

Layer Number	Type	Feature Map Number	Convolutional Kernel Size	Feature Map Size	Neuron Number
1	Convolutional Layer	6	5 × 5	28 × 28	4704
2	Pooling Layer	6	2 × 2	14 × 14	1176
3	Convolutional Layer	12	5 × 5	10 × 10	1200
4	Pooling Layer	12	2 × 2	5 × 5	300
5	Fully-connected Layer	120	1 × 1	1 × 1	120
6	Fully-connected Layer	84	1 × 1	1 × 1	84
7	Output Layer	43	-	-	43

**Table 3:** The classification and recognition test results of six categories of traffic signs.

Sequence Number	Traffic Signs Type	Test Images Number	TP	FN	Accurate Recognition Rate (%)	Average Processing Time (ms)/Frame
1	Speed Limit	1000	997	3	99.70	5.4
2	Danger	1000	999	1	99.90	5.8
3	Mandatory	1000	997	3	99.70	5.2
4	Prohibitory	1000	998	2	99.80	4.9
5	Derestriction	1000	994	6	99.40	6.4
6	Unique	1000	1000	0	100.00	4.7
Total	-	6000	5985	15	99.75	5.4

**Table 4:** The comparison of statistics in algorithm performance based on the GTSRB dataset.

Serial Number	Method	Accurate Recognition Rate (%)	Average Processing Time (ms)/Frame	System Environment
1	Multilayer Perceptron [39]	95.90	5.4	Intel Core i5 processor, 4 GB RAM
2	INNLP + INNC [40]	98.53	47	Quad-Core AMD Opteron 8360 SE, CPU
3	GF+HE+HOG+PCA [41]	98.54	22	Intel Core i5 processor @2.50 GHz, 4 GB RAM
4	Weighted Multi-CNN [42]	99.59	25	NVIDIA GeForce GTX 1050 Ti GPU, Intel i5 CPU
Ours	Proposed Method	99.75	5.4	Intel(R) Core(TM) i5-6500 CPU @3.20GHz
